# Supplementary figures and images for: Impact response of lightweight steel foam concrete composite slabs: Experimental, numerical and analytical studies
Source: PLoS One. 2024 Jan 12;19(1):e0296303. doi: 10.1371/journal.pone.0296303 (PMC10786397; doi:10.1371/journal.pone.0296303)

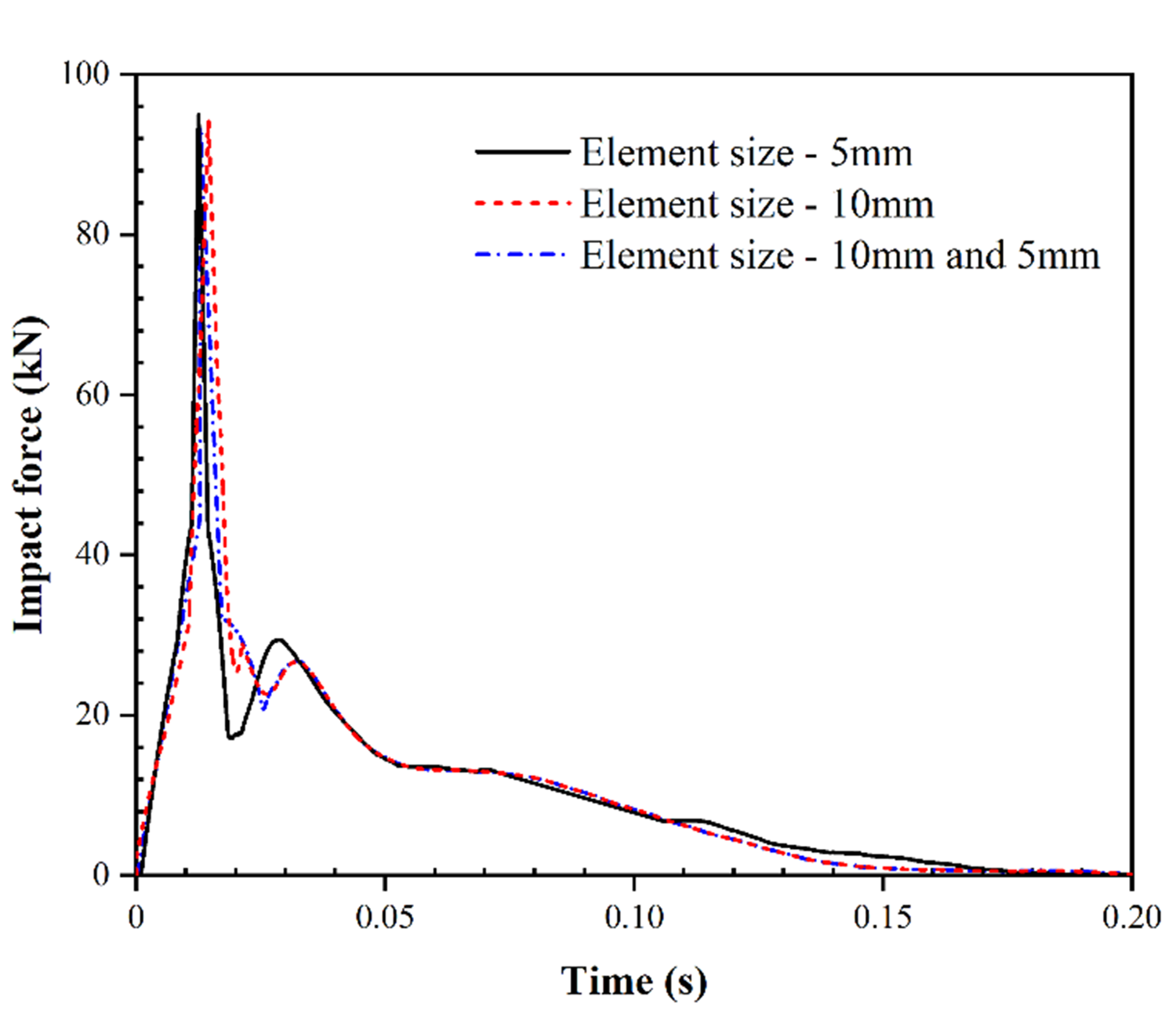

Supplement: S1 Fig — (TIF) [file pone.0296303.s001.tif]

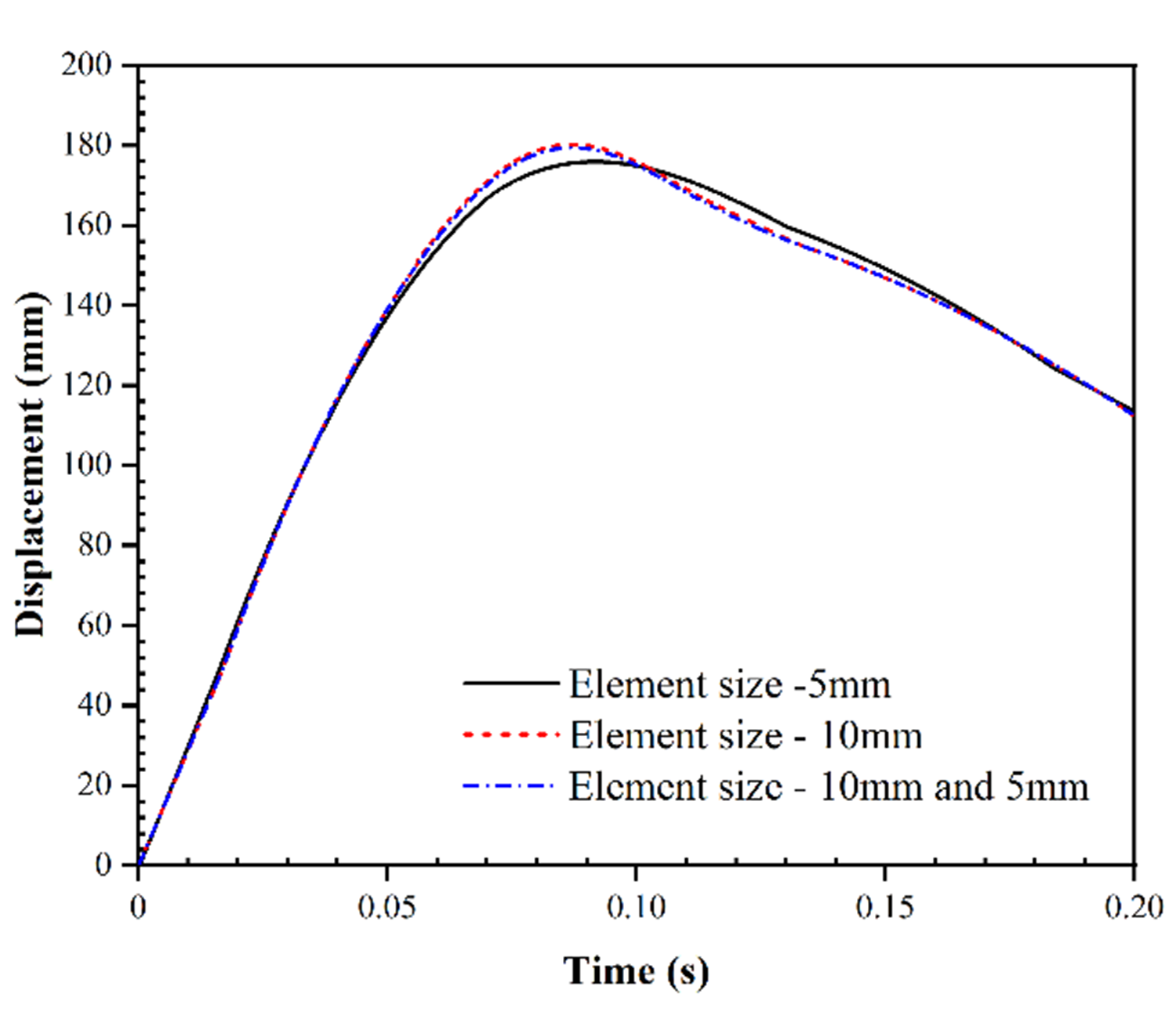

Supplement: S2 Fig — (TIF) [file pone.0296303.s002.tif]
